# Application of grey feed forward back propagation-neural network model based on wavelet denoising to predict the residual settlement of goafs

**DOI:** 10.1371/journal.pone.0281471

**Published:** 2023-05-04

**Authors:** Xiangdong Zhang, Wenliang Li, Xuefeng Zhang, Guanjun Cai, Kejing Meng, Zhen Shen

**Affiliations:** 1 School of Civil Engineering, Liaoning Technical University, Fuxin, Liaoning, China; 2 Beijing Jingneng Geological Engineering Co., Ltd, Beijing, China; National Institute of Technology, India (Institute of National Importance), INDIA

## Abstract

To study the residual settlement of goaf’s law and prediction model, we investigated the Mentougou mining area in Beijing as an example. Using MATLAB software, the wavelet threshold denoising method was used to optimize measured data, and the grey model (GM) and feed forward back propagation neural network model (FFBPNN) were combined. A grey feed forward back propagation neural network (GM-FFBPNN) model based on wavelet denoising was proposed, the prediction accuracy of different models was calculated, and the prediction results were compared with original data. The results showed that the prediction accuracy of the GM-FFBPNN was higher than that of the individual GM and FFBPNN models. The mean absolute percentage error (MAPE) of the combined model was 7.39%, the root mean square error (RMSE) was 49.01 mm, the scatter index (SI) was 0.06%, and the BIAS was 2.42%. The original monitoring data were applied to the combination model after wavelet denoising, and MAPE and RMSE were only 1.78% and 16.05 mm, respectively. Compared with the combined model before denoising, the prediction error was reduced by 5.61% and 32.96 mm. Thus, the combination model optimized by wavelet analysis had a high prediction accuracy, strong stability, and accorded with the law of change of measured data. The results of this study will contribute to the construction of future surface engineering in goafs and provide a new theoretical basis for similar settlement prediction engineering, which has strong popularization and application value.

## Introduction

To perfect new urbanization strategies for accelerated development, utilizing goaf sites as a building foundation has become an important measure to solve the problem of land shortage [1, 2]. Therefore, it is necessary to establish a prediction model to predict the residual settlement of old goaf surfaces and ensure the safety and stability of new buildings on these surface [3–5].

Among the prediction models proposed by scholars, the grey model (GM) and feed forward back propagation neural network (FFBPNN) model prediction methods are the most widely used to predict surface subsidence in underground mining areas [6–8]. In 1982, Professor Deng, a Chinese scholar, proposed the grey system theory integrating automatic control and operations research, aiming at the in-depth exploration of grey problems [9]. Xu et al. used remote sensing interpretation data and measured data as data sources. The GM (1,1) was used to dynamically predict the ground subsidence of goafs, and the results showed that the maximum residual between the predicted and actual value was only 2.4 mm. The correlation coefficient (R^2^) was greater than 0.95, suggesting a good degree of curve fitting [10]. Due to the uncertainty of mining subsidence, Xu et al. established the row vector average sequence GM (1,1), column vector average sequence GM (1,1), and cell volume sequence GM (1,1) to model and analyse the monitoring data, for practical verification, and for model accuracy, respectively [11]. Furthermore, Wang et al. conducted a comparison test of various GMs to determine the nonlinear change of residual settlement in goafs. They found that the predicted values of the discrete DGM (1,1) and the GM-Markov models were closer to the actual values than the traditional GM model, with higher stability [12, 13].

In 1986, American scientists, Rumelhart and McClelland, put forward the concept of back propagation neural networks based on artificial neural network (ANN) algorithms using nonlinear neuron processing functions. This is a multilayer feed forward network trained by an error back propagation algorithm; its advantages lay in its simple structure, flexibility, and convenience, making it suitable for the study of nonlinear problems, such as surface subsidence caused by coal mining [14]. The artificial intelligence model is also widely used in other fields of civil engineering [15, 16]. Lee et al. combined this artificial neural network model with the geographic information system to evaluate and predict land subsidence changes in an abandoned coal mine in South Korea, based on the existing land subsidence information. They verified that the subsidence development trend predicted by ANN was consistent with actual conditions [17]. Pei et al. used a genetic algorithm to optimise the parameters of the BP neural network model, using 36 training sample groups and 4 test sample groups for analysis. Their results showed that the prediction was consistent with actual engineering conditions, suggesting that this model has a significant role in the study of nonlinear change problems with randomness [18]. The surface subsidence value predicted by Li et al. using the GA-BP neural network model was compared with the monitoring value obtained by PS-InSAR technology, and the deviation between both results was found to be within a reasonable range, thereby verifying the feasibility and accuracy of the GA-BP neural network model for predicting ground subsidence [19]. Based on Fourier analysis, wavelet analysis can effectively distinguish unstable signals, refine signals, and analyse the local situation of the signal. Wavelet transform weakens the signal noise and restores the reconstructed signal; therefore it is widely used in engineering practice [20, 21].

The GM and BP neural network model are more effective at predicting series changes; however, there are limitations. The disadvantage of the GM is that the mathematical formula by which it deals with error and nonlinear fitting is not ideal. Similarly, the BP neural network model has a slow convergence speed and high demand for data samples. With the continuous expansion of their application, the shortcomings and deficiencies of single prediction models are gradually being highlighted [22–24]. In this study, the grey feed forward back propagation neural network prediction mechanism based on wavelet theory was used to predict and analyse the residual settlement of the old mined-out area in the Mentougou mining area in Beijing. This combination model provides a new basis for the theoretical exploration and practical application of surface deformation monitoring and prediction engineering in mining areas.

## Method

### Grey prediction model

1. Data monitoring in practical engineering is usually limited due to short monitoring cycles, therefore, it is necessary to adopt a targeted prediction method to study the settlement and deformation trend of goafs [25]. Grey prediction models require little historical data to predict unknown information, and the GM (1,1) is most commonly used as a single-sequence first-order grey linear model. Its modelling process is as follows: (1) The original data sequence is established:

$$x^{\left(0\right)}=[x^{\left(0\right)}(1),x^{\left(0\right)}(2),\cdots ,x^{\left(0\right)}(n)]$$ (1)

Stepwise accumulation (1-AGO) generates a new prediction sequence:

$$x^{\left(1\right)}=[x^{\left(1\right)}(1),x^{\left(1\right)}(2),\cdots ,x^{\left(1\right)}(n)]$$ (2)

$$x^{\left(1\right)}(t)=\sum_{i=1}^{t}x^{\left(0\right)}(i),t=1,2,\cdots ,n$$ (3)

(2) Grey differential equations for cumulative sequences are established:

$$x^{\left(0\right)}(t)=az^{\left(1\right)}(t)=b,t=2,3,\cdots ,n$$ (4)

Where *z*^(1)^ represents adjacent mean sequence generated for *x*^(1)^:

$$z^{\left(1\right)}=[z^{\left(1\right)}(2),z^{\left(1\right)}(3),\cdots ,z^{\left(1\right)}(n)]$$ (5)

$$z^{\left(1\right)}(t)=0.5x^{\left(1\right)}(t)+0.5x^{\left(1\right)}(t-1)$$ (6)

The corresponding whitening differential equation:

$$\frac{dx^{\left(1\right)}}{dt}+ax^{\left(1\right)}=b$$ (7)

In the formula *a* is the development coefficient and *b* is the grey action.

(3) Coefficient matrix *B* and constant term *Y* are constructed, *a* and *b* are calculated via the least square method:

$$B=[\begin{array}{cc} -z^{\left(1\right)}(2) & 1\\ -z^{\left(1\right)}(3) & 1\\ \cdot & \cdot \\ \cdot & \cdot \\ \cdot & \cdot \\ -z^{\left(1\right)}(n) & 1 \end{array}],Y=[\begin{array}{c} x^{\left(0\right)}(2)\\ x^{\left(0\right)}(3)\\ \cdot \\ \cdot \\ \cdot \\ x^{\left(0\right)}(n) \end{array}]$$ (8)

$$u={\left(a,b\right)}^{T}={\left(B^{T}B\right)}^{-1}B^{T}Y$$ (9)

(4) The values of *a* and *b* are substituted back into the original differential equation and the time response formula of the grey differential is derived:

$$x^{\left(1\right)}(t+1)=(x^{\left(0\right)}(1)-\frac{b}{a})e^{-at}+\frac{b}{a}$$ (10)

The prediction model of the original sequence can be obtained according to the following formula:

$$x^{\left(0\right)}(t+1)=x^{\left(1\right)}(t+1)-x^{\left(1\right)}(t)=(1-e^{a})(x^{\left(0\right)}(1)-\frac{a}{b})e^{-at}$$ (11)

### FFBPNN model

**Fig 1:**
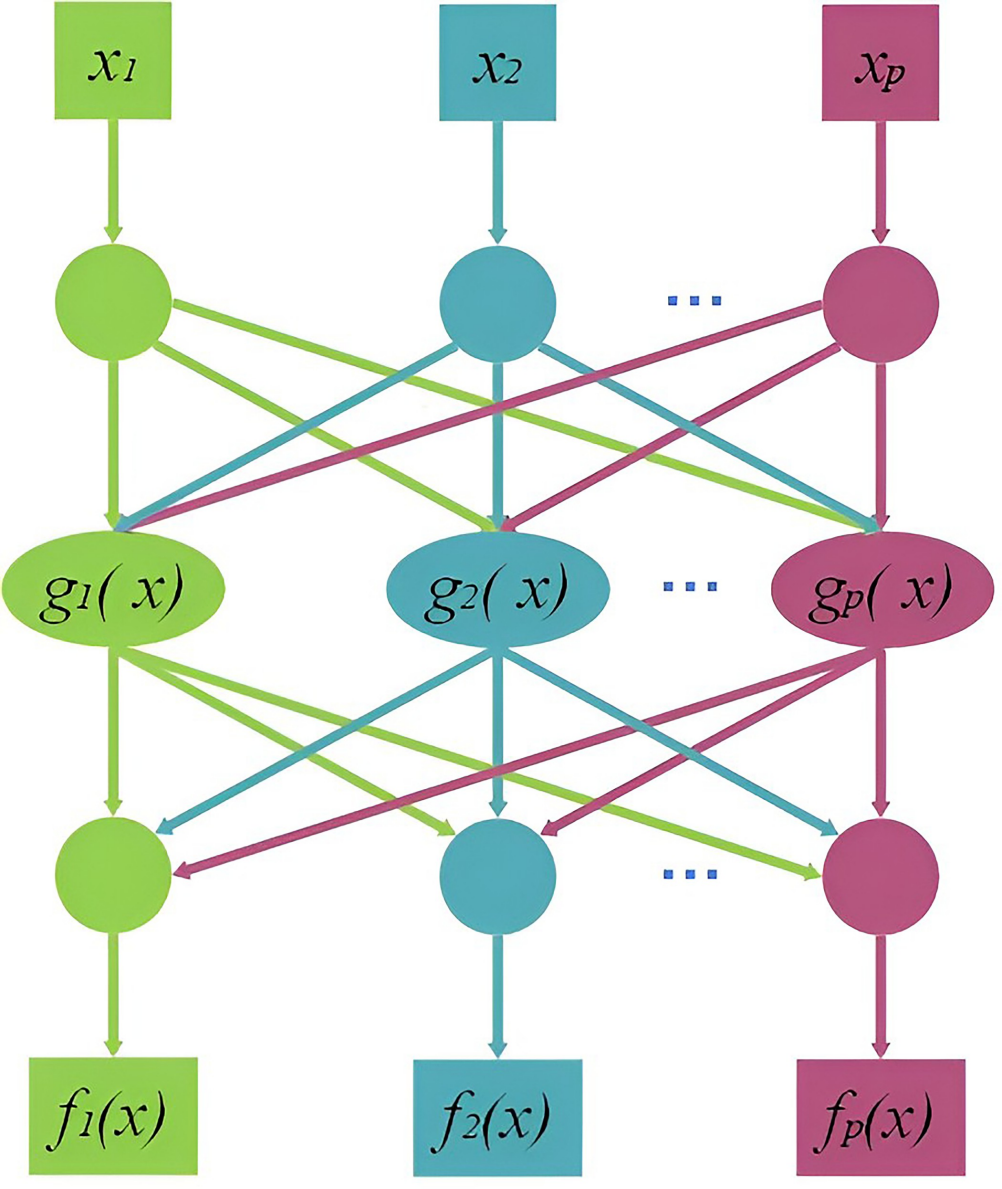
FFBPNN structure.

**Fig 2:**
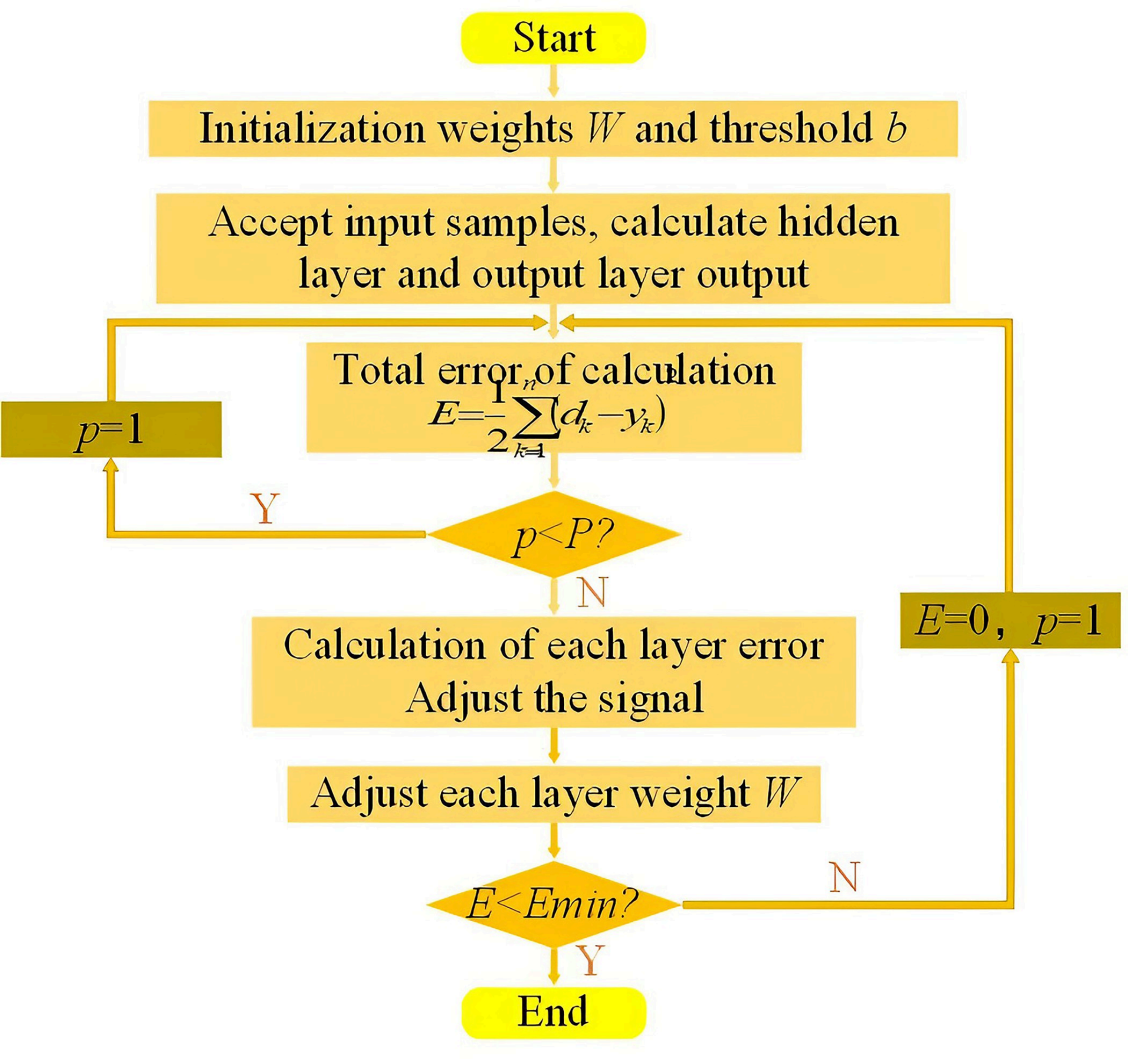
Flow chart of FFBPNN.

The Artificial Neural Network (ANN) is a widely used information processing technology, and the FFBPNN is particularly effective for data prediction and is better suited for dealing with changes in surface nonlinear subsidence caused by coal mining [26]. The FFBPNN has a strong learning ability and large storage space for mapping the relationship between input and output patterns. Its structure is not restricted, but is typically divided into the input, hidden, and output layers. The neurons in each layer are independent of each other and connected between the layers, as shown in Figs 1 and 2.

### GM-FFBPNN prediction model

**Fig 3:**
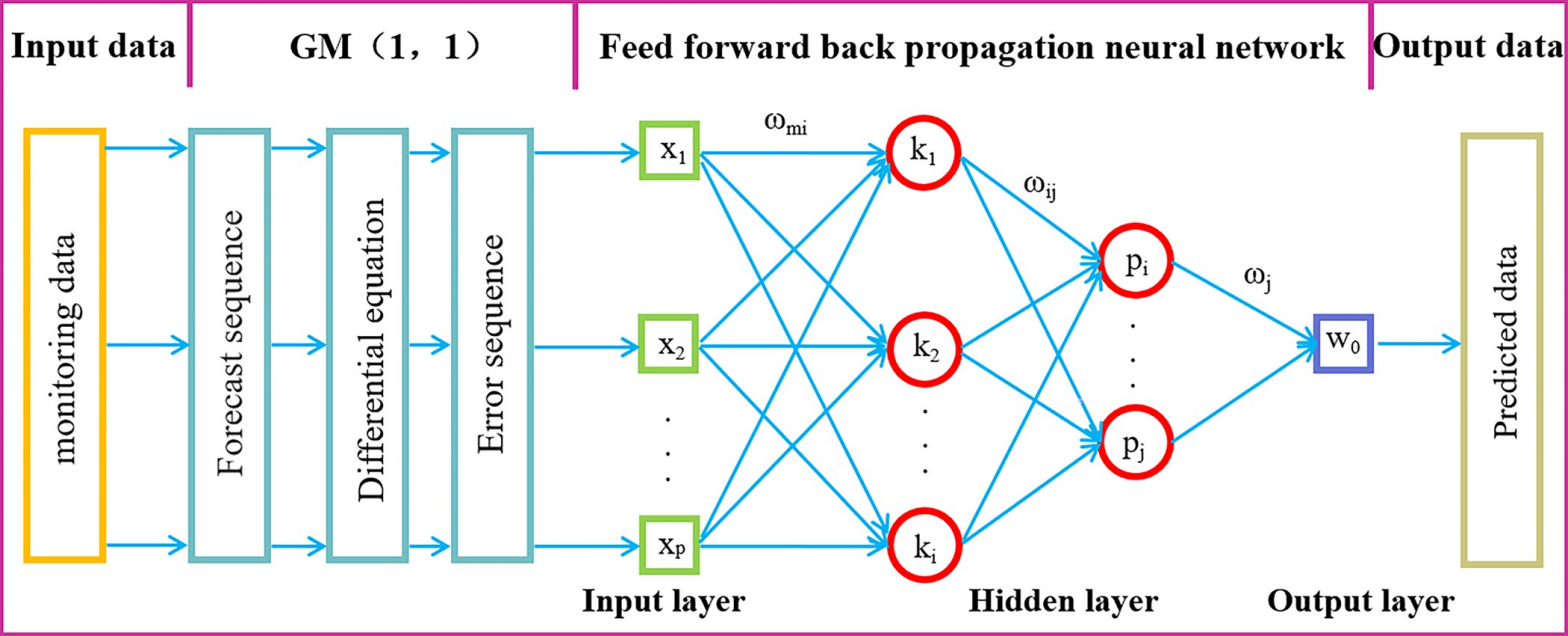
Prediction flow chart of GM-FFBPNN model.

To avoid the shortcomings of using a single model, we serially combined the GM (1,1) and FFBPNN models, which were chosen for their excellent individual performances, to build a GM- FFBPNN model [27]. The combined model was used to make a preliminary prediction of the data sequence usin the GM (1,1), which was then used as the learning samples for further prediction by the FFBPNN and acquisition of the error sequence of the combination model. The specific prediction process is depicted in Fig 3. The combined model effectively reduced the error and improved the prediction accuracy, the specific modelling process is as follows:

(1) Based on the raw data sequence, $x^{\left(0\right)}=[x^{\left(0\right)}(1),x^{\left(0\right)}(2),\cdots ,x^{\left(0\right)}(n)]$, the GM (1,1) was used for prediction, and the prediction sequence was ${\hat{x}}^{\left(0\right)}=[{\hat{x}}^{\left(0\right)}(1),{\hat{x}}^{\left(0\right)}(2),\cdots ,{\hat{x}}^{\left(0\right)}(n)]$.
(2) The error sequence, $\varepsilon^{\left(0\right)}=x^{0}-{\hat{x}}^{0}$, was obtained by subtracting e original data sequence *x*^(0)^ from the prediction sequence ${\hat{x}}^{\left(0\right)}$.
(3) Taking the prediction sequence ${\hat{x}}^{\left(0\right)}$ and error sequence *ε*^(0)^ as the input and output samples, respectively, the FFBPNN model was trained to obtain the corresponding weight *W* and threshold *b*.
(4) The error sequence *ε*^(0)^ was imputed into the trained BP neural network model for further prediction and to obtain the new error sequence *ε*’^(0)^.
(5) The prediction sequence ${\hat{x}}^{0}$ and the new error sequence *ε*’^(0)^ were added to obtain the prediction value $Z^{\left(0\right)}={\hat{x}}^{\left(0\right)}+\varepsilon^{'(0)}$ of the GM-FFBPNN model.

Comparing the original data with the predicted value, the prediction accuracy of the combined model was calculated and evaluated.

### The basic principle of wavelet denoising

The signal of the original monitoring data had to fluctuate noise signal, affecting the real monitoring information and accuracy of derived ground subsidence data. When dealing with such nonlinear signals, wavelet transform can reduce or eliminate random signals, extract system signals, and provide more accurate data support for deformation predictions [28].

The wavelet transforms of any continuous function signal *f*(*t*) is defined as:

$$WT_{f}(a,WT_{f}(a,b)=|a{|}^{-1/2}\int_{-\infty }^{\infty }f(t)\psi (\frac{t-b}{a})dt=\langle f,\psi_{a,b}\rangle a\neq 0$$ (12)

Where $\psi_{a,b}(t)={\left|a\right|}^{-1/2}\psi (\frac{t-b}{a})$ make the contravariant transformation exist, *ψ*(*t*) needs to meet the admissible row condition:

$$C_{\psi }=\int_{-\infty }^{\infty }\frac{{\left|\hat{\psi }(\omega )\right|}^{2}}{\left|\omega \right|}d\omega <\infty$$ (13)

Where $\hat{\psi }(\omega )$ is the Fourier transform of *ψ*(*t*), then the inverse transform can be calculated as:

$$f(t)=C_{\psi }^{-1}\int_{-\infty }^{\infty }\int_{-\infty }^{\infty }\psi_{a,b}(t)WT_{f}(a,b)db\frac{da}{|a{|}^{2}}$$ (14)

The field of wavelet theory has facilitated the exploration of a more mature and perfect theoretical system due to its wide application, from which the wavelet threshold denoising method has been gradually developed. The method is flexible and accurate, and the principle is simple. It can effectively remove noise and retain real signal characteristics and has a wide range of applications in many fields. Normally, the frequency of the real signal is low while that of noise is high. Thus, the principle of wavelet threshold denoising is to reduce or remove the noise distributed in the high-frequency wavelet coefficients. The one-dimensional signal model contains noise as follows:

s(n)=f(n)+σe(i),i=1,2,⋯,n−1 (15)

Where *s*(*n*) represents the monitoring signal, *f*(*n*) is the real signal, *σ* denotes the noise level, and *e*(*i*) indicates the noise signal.

## Results and discussion

### Geological conditions and monitoring

**Fig 4:**
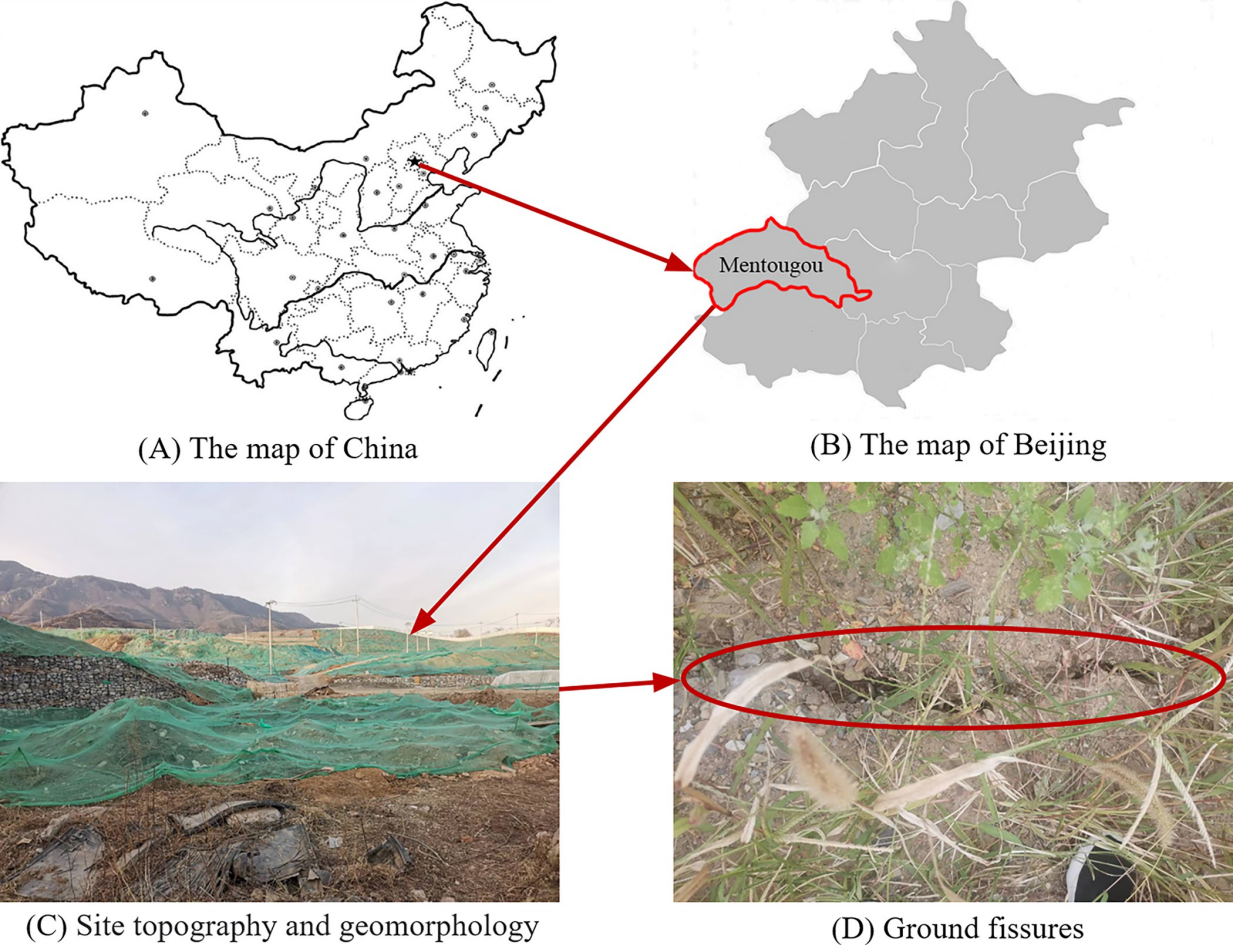
Study area: (A) The map of China; (B) The map of Beijing; (C) Site topography and geomorphology; (D) Ground fissures.

The planned land is in Longquan Town, Mentougou District, Beijing, and the geological structure of the site is medium complex, as shown in Fig 4A and 4B. The area has low relief and simple landform types, as shown in Fig 4C. The proposed site was located above the #9 coal seam of the Mentougou minefield. On-site data collection and visits to the surrounding residents revealed that the shallow surrounding the field is a historical small coal mining site. Most of these coal mines were mined privately or collectively using the basic room-and-pillar coal mining method, with a low recovery rate. The mining depth was generally no more than 60 m, and the dip angle was 6–8°, which is a gently inclined coal seam. After coal seam mining, goaf overburden movement induces uneven surface settlement, which increases the number of surface fissures, as shown in Fig 4D.

**Fig 5:**
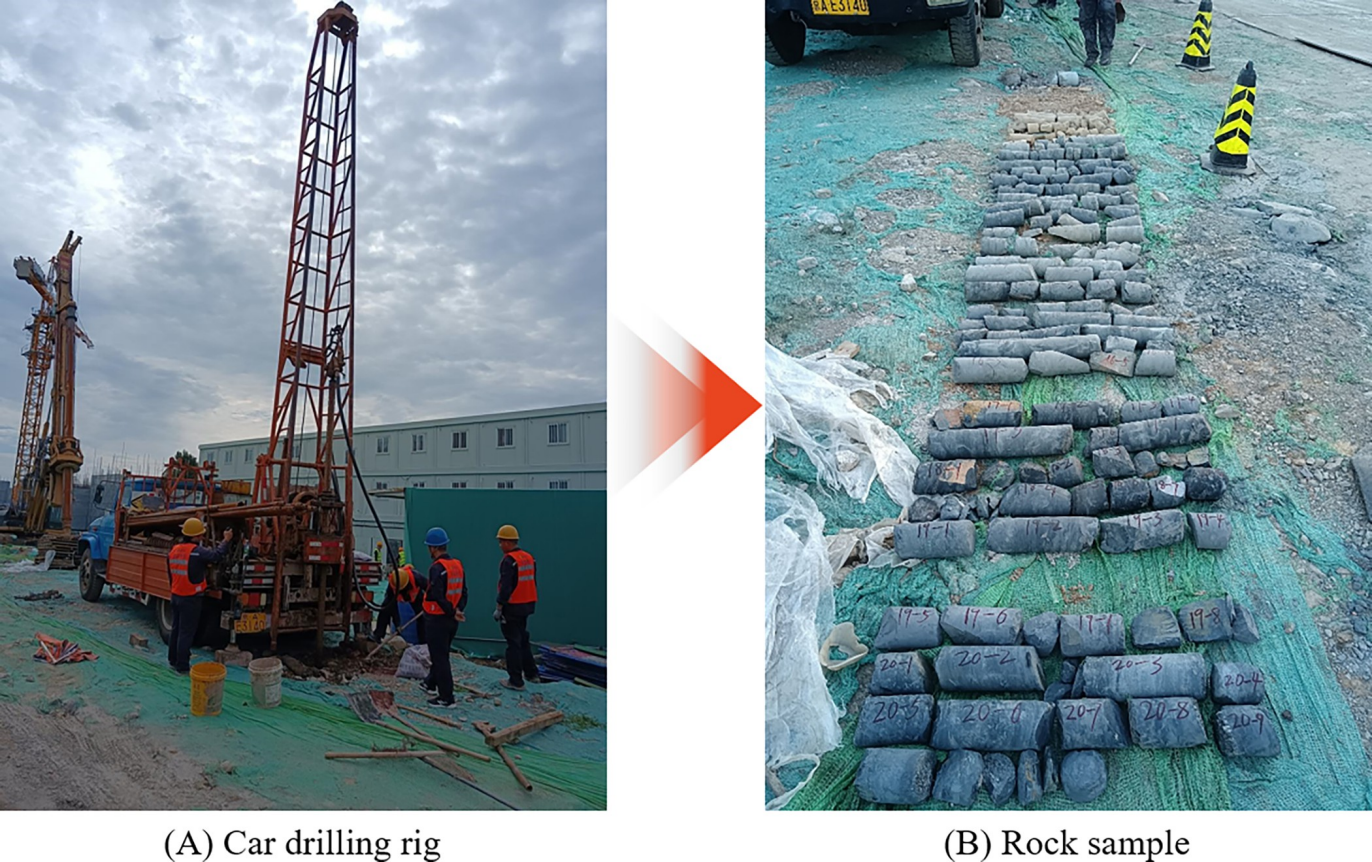
Drilling test: (A) Car drilling rig; (B) Rock sample.

The DPP-100 car drilling rig was used for the geological survey to determine the engineering geological conditions in the proposed site. The car drilling rig and some rock samples are shown in Fig 5. Based on drilling samples, *in-situ* testing, and geotechnical test results, the strata in this area were divided into five layers, according to rock and soil characteristics. The first layer was an artificial filling soil layer, the second layer was a general Quaternary sedimentary layer that was mainly composed of silty clay and gravel, and the third to fifth layers were sandstone and coal seams of different weathering degrees. Considering the shallow mining depth of the small coal kiln without support measures, the area was presumed to be at risk of ground collapse.

**Fig 6:**
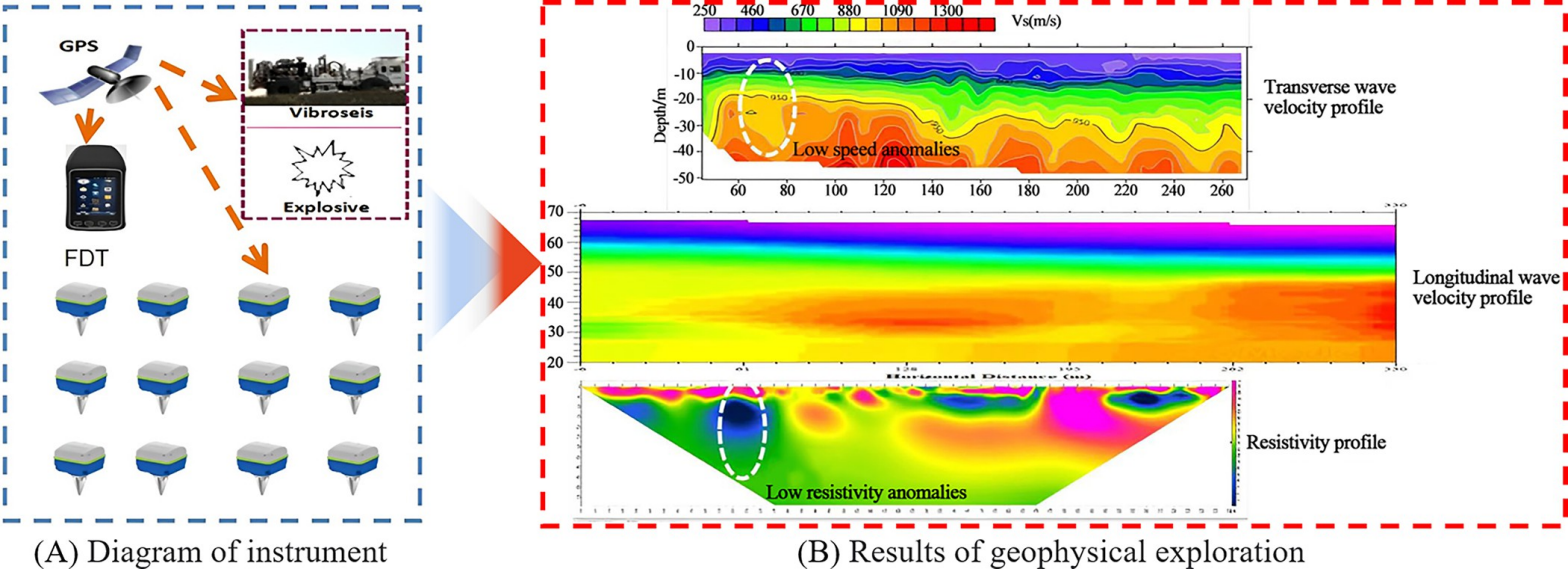
Geophysical exploration test: (A) Diagram of instrument; (B) Results of geophysical exploration.

The Smartsolo IGU-16 nodal seismograph was used for physical detection in the study area. The instrument performs automatic sensor detection and GPS positioning, as well as efficient data collection. According to a comprehensive analysis of the geophysical exploration results, the physical characteristics of individual points were abnormal and preliminarily confirmed as goaf. In conjunction with drilling data verification, the specific burial depth and development characteristics of the underlying goaf were determined, as shown in Fig 6.

**Fig 7:**
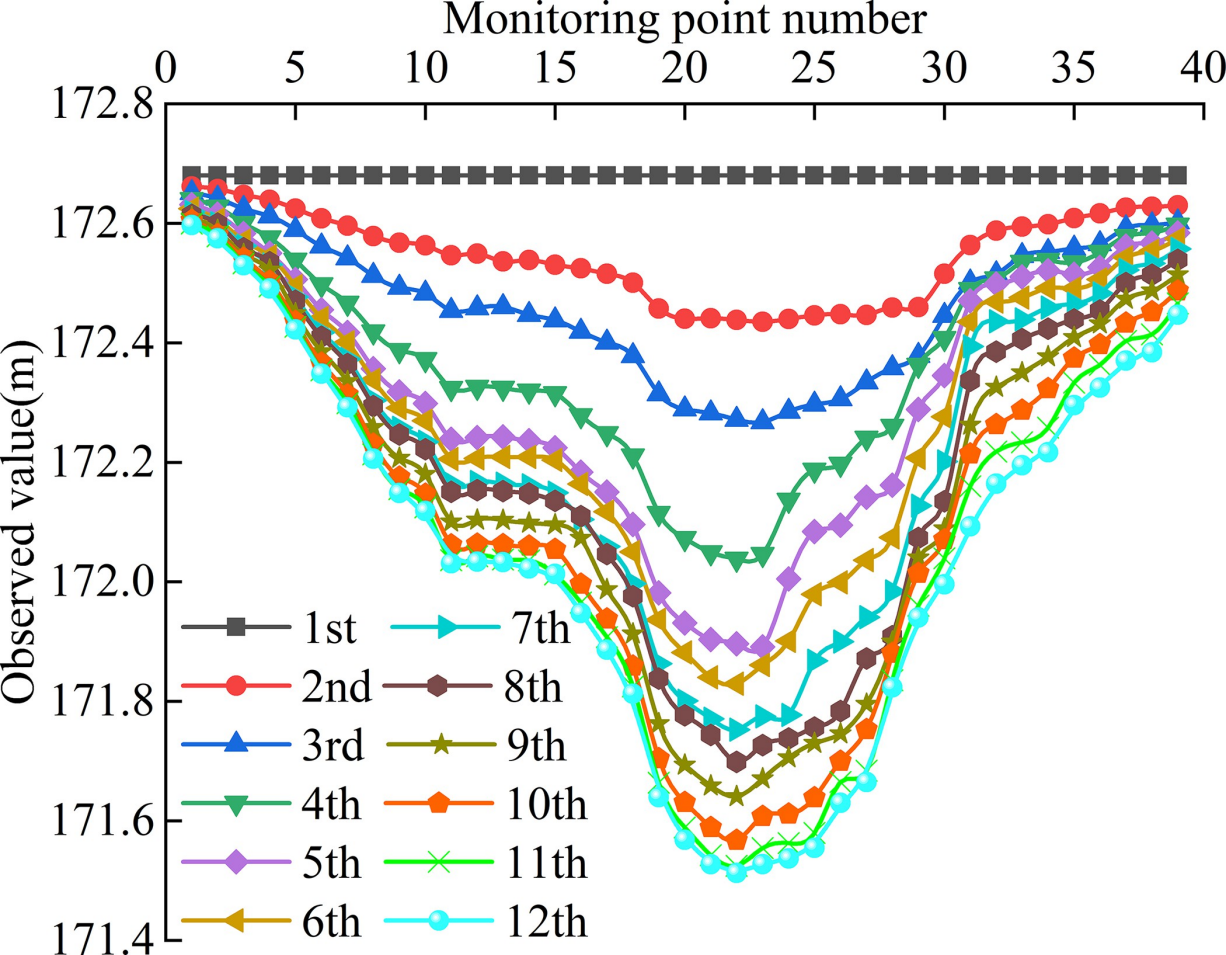
The observation curve of monitoring points.

To study the basic law of surface residual settlement deformation after coal mining, surface subsidence data were obtained via field measurement. Due to the terrain constraints and a shortage of human resources in the early stages, only one inclination observation line was established from east to west on the south side of the planned area. There were 39 monitoring points (N1–N39), and the interval between the two monitoring sessions was about 1 year, with a total of 12 monitoring sessions recording work performed using observation stations in accordance with the relevant provisions of the International Organization for Standardization [29]. Lines were drawn based on the data of rock movement that was monitoring the curves of monitoring points on the trend observation (Fig 7).

As shown in Fig 7, the overall change process of surface movement and deformation was continuous and gradual, presenting an asymmetrical distribution. Over time, the surface observation values from the monitoring points on both sides of the east and west to the central goaf showed a decreasing trend. The subsidence basin was mainly concentrated on the surface above the goaf and the curve shape conformed to the general law of surface subsidence. By computing the cumulative subsidence of stage 12 at point N22 near the goaf boundary, the maximum settlement was determined to be 1,166.9 mm, the average annual subsidence was 106.1 mm, and the settlement value of the easternmost monitoring point N1 was the lowest at 82.2 mm, indicating that the surface was still settling. The measured data of N22 monitoring points are shown in (Table 1).

**Table 1:** The measured data of N22 monitoring points.

Observation period	Observed value (m)	Cumulative subsidence value (mm)	Observation period	Observed value (m)	Cumulative subsidence value (mm)
1	172.68	0.0	7	171.75	-927.6
2	172.44	-241.7	8	171.70	-981.3
3	172.27	-408.5	9	171.64	-1039.3
4	172.04	-641.7	10	171.57	-1112.4
5	171.90	-783.8	11	171.52	-1155.3
6	171.83	-850.6	12	171.51	-1166.9

**Fig 8:**
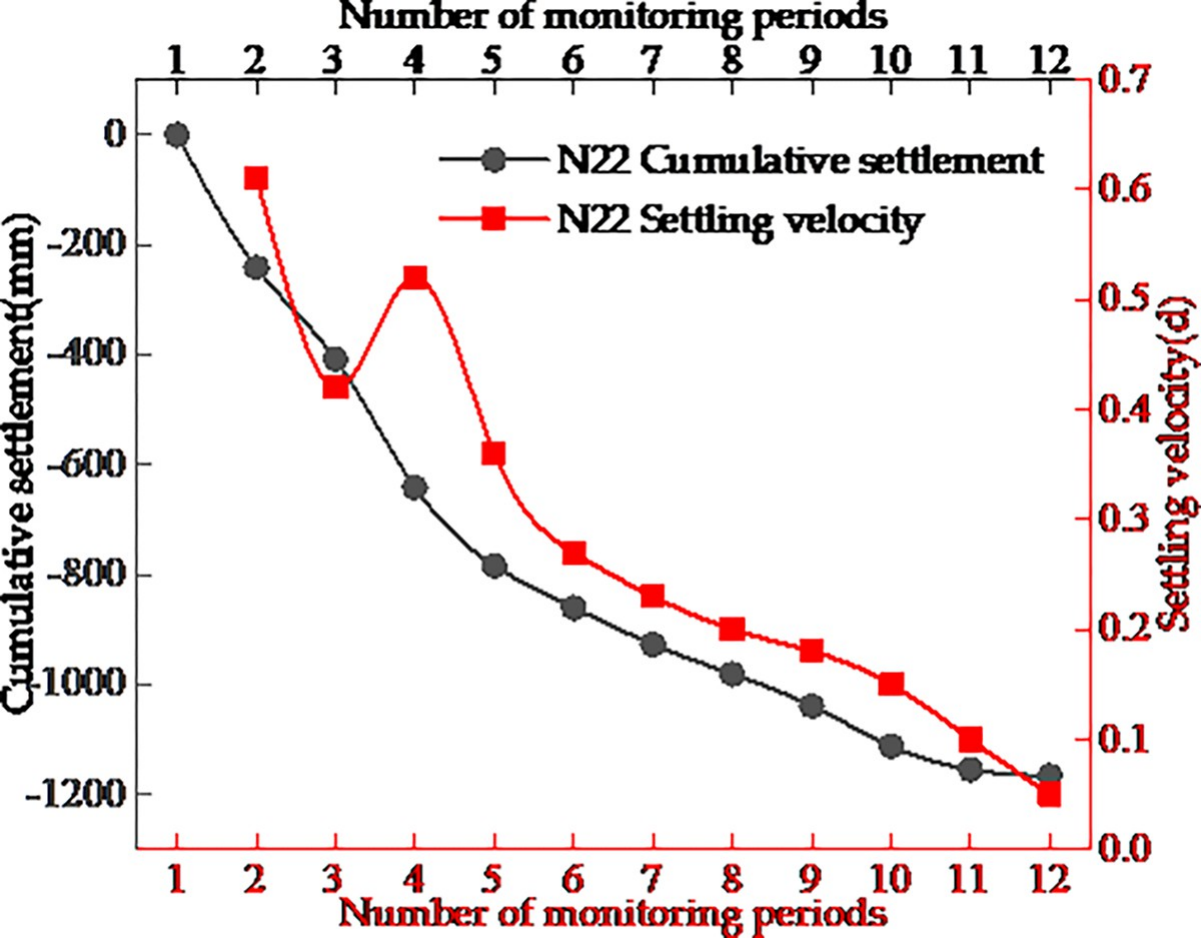
Cumulative settlement and settlement velocity curve of N22 monitoring points.

As mining in the Mentougou coal mine and other small coal mines has ceased, the surface above has undergone rapid deformation and is now in the residual deformation stage [30]. According to the observation data, the cumulative settlement curve and settlement velocity curve of the maximum subsidence point N22 monitoring site during the monitoring period were calculated and drawn (Fig 8). The accumulated settlement of N22 point increased gradually with monitoring time, whereas the decline curve appeared to be gentle, presenting a slow semi-parabolic downward trend. The settlement velocity curve of the N22 point generally showed a trend of slow decrease, but appeared to have an inflection point, thus it was not in conformity with general laws. This may be because the shanty towns established near the monitoring points, as well as new buildings and human activities, have increased the load on the surface of the goaf, resulting in a sudden increase in sinking speed followed by a gradual decrease. The maximum subsidence velocity of monitoring point N22 reached 0.61 mm/d, less than the subsidence speed during the active period of surface movement (1.7 mm/d) stipulated in The Code for Coal Pillar Establishment and Coal Pressing Mining of Buildings, Water Bodies, and Railways and Main Shafts and Lanes [31]. During this time, the subsidence process was gentle and in the recession period of surface movement, which little influence on buildings.

### GM (1,1) model prediction

According to the principle of maximum subsidence, the actual settlement date of the N22 monitoring point on the goaf surface observation line was selected as the original sequence, generating the calculation sequence using a one-time accumulation method, by establishing the first-order linear differential equation to solve the development coefficient *a* and the grey action *b*. The fitting GM (1,1) prediction model of the accumulated settlement of the N22 point was obtained by substituting the original differential equation, and the prediction accuracy was tested [32] (Table 3). Subsequently, the original and predicted values were compared and analysed to obtain residual and relative errors predicted by the model (Table 3).

Table 2 shows that the posterior error ratio of the GM (1, 1) prediction model was 0.0249 < 0.35, and the small error probability value was 1 > 0.95, indicating that the model had a high level of precision that met the first level prediction precision standard [33]. Comparing the forecast data of the GM (1,1) with the original data, the overall fitting degree was high, but the residual and relative errors of individual prediction values were large. This is due to the limited ability of the grey theory to use and process deterministic information, as well as its failure to adjust the error feedback in time, resulting in large and uncontrollable errors. Meanwhile, the relative error ranged from -39.26% to 12.02%, the prediction results were heterogeneous, and error polarization occurred, indicating that the prediction results of the single model were not very accurate. Solving nonlinear field problems using the prediction model established by grey theory alone was difficult. Therefore, other prediction models should be combined to reduce error and improve prediction accuracy.

**Table 2:** Model building results.

Development coefficient	Grey action	Fitting GM (1,1) model	Posterior error ratio	Probability of small error
0.0772	1931.9822	${\hat{x}}^{\left(1\right)}(t+1)=-25267.4e^{-0.0772t}+25025.7$	0.0249	1

### FFBPNN model prediction

**Fig 9:**
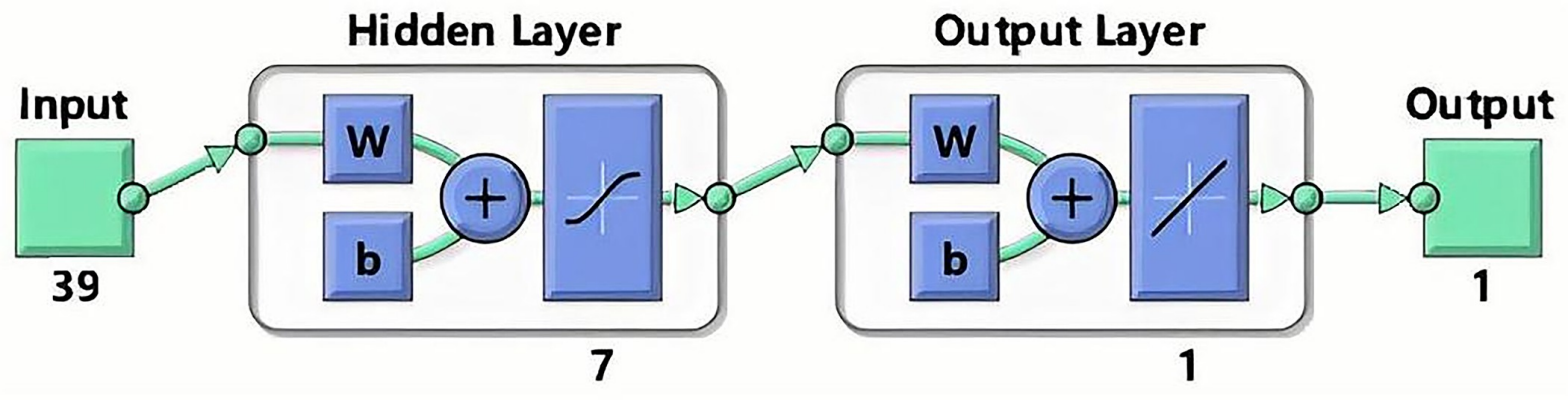
FFBPNN structure diagram.

**Fig 10:**
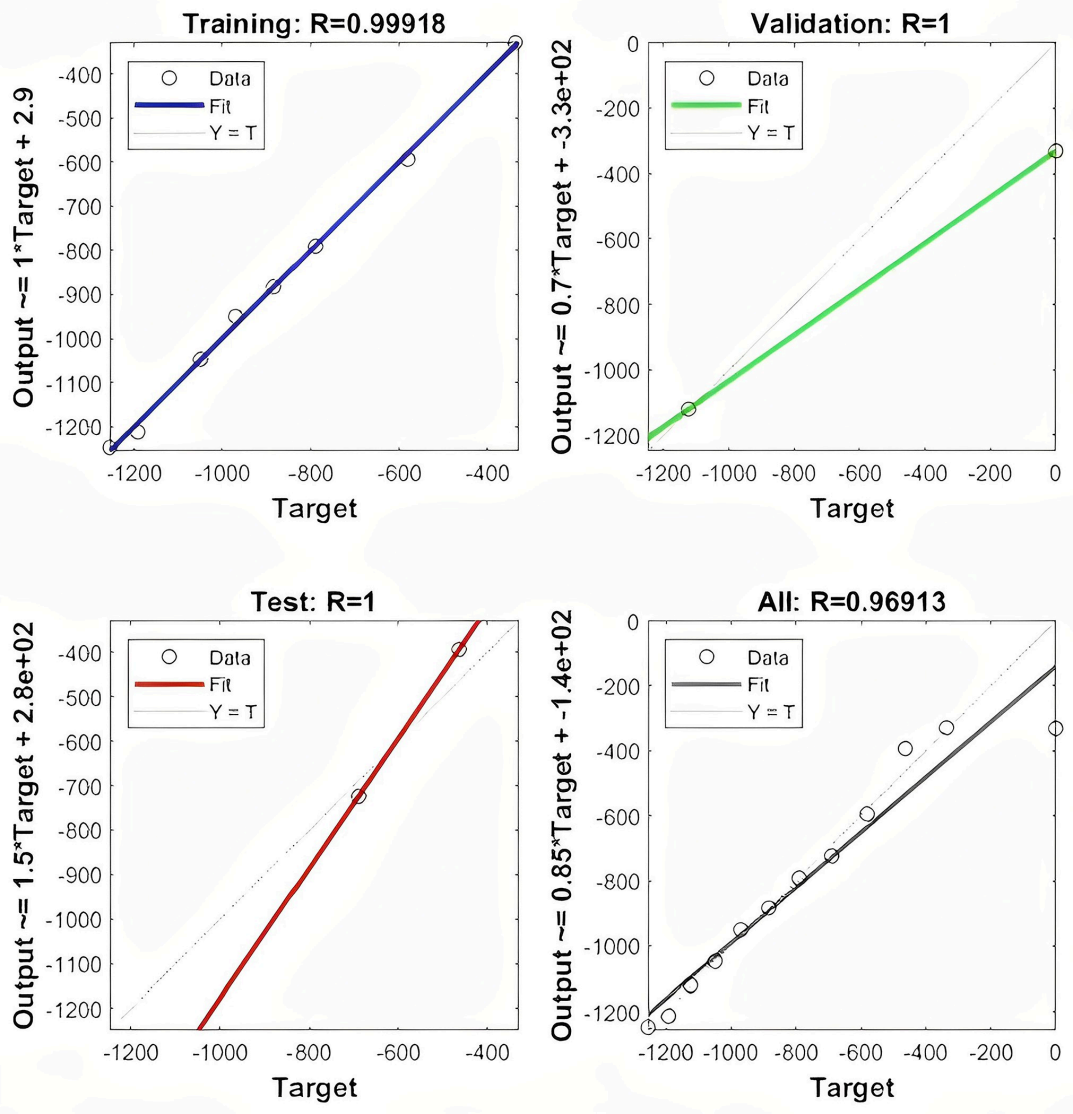
Fitting regression diagram of subsidence prediction model.

From the predicted results of the GM (1,1), we found that the prediction error of the model for the cumulative settlement of the initial monitoring point was large, therefore, the MATLAB R2021a software programming method was used to achieve multiple training of samples to reduce the error of the predicted value. First, the classical three-layer topology structure was selected to build the FFBPNN prediction model, and ground subsidence data from one of the goafs in this project was used as input for training. The number of nodes in the input layer was *i* = 39, the number of nodes in the output layer was *k* = 1. According to the empirical formula: $j=\sqrt{i+k}+\alpha ;\alpha \in (1,10)$, the number of hidden layer nodes was set to 7. The Levenberg-Marquardt algorithm with high accuracy and fast convergence speed was used to calculate and complete the modeling. As shown in Fig 9. Following the construction of the FFBPNN model, iterative training was performed, the neural network model converged after 7 iterations, with an overall R of 0.99689. All data points were evenly distributed near the fitting curve, indicating that the model had a high fitting degree and strong prediction ability, as shown in Fig 10. To verify the accuracy of this model, it was applied to the built-in data set of MATLAB software for testing. This data set was large and popular, confirming the correctness and accuracy of the constructed model. During the FFBPNN model training process, the data set was divided into training, validation, and test sets in the ratio of 70%:15%:15% [34].

### GM-FBPNN model prediction

To combine the advantages of both models, the fitting results of the GM (1,1) were used as input values for neural network prediction, and the error sequence was then imputed into the trained FFBPNN prediction model. The modified error sequence was added to the predicted sequence of the GM (1,1), which was the predicted value of the GM-FFBPNN (Table 3).

**Table 3:** Comparison between original and predicted values of the two models.

Observation period	Original value (mm)	GM (1, 1)			FFBPNN			GM-FFBPNN		
		Predicted value (mm)	Residual error (mm)	Relative error (%)	Predicted value (mm)	Residual error (mm)	Relative error (%)	Predicted value (mm)	Residual error (mm)	Relative error (%)
1	0.0	0.0	0.0	0.00	-0.7	0.7	**	-1.3	1.3	**
2	-241.7	-336.6	94.9	-39.26	-318	76.3	-31.57	-296.7	55.0	-22.76
3	-408.5	-463.5	55.0	-13.46	-466.5	58.0	-14.20	-470.2	61.7	-15.10
4	-641.7	-580.9	-60.8	9.48	-584	-57.7	8.99	-587.3	-54.4	8.48
5	-783.8	-689.6	-94.2	12.02	-703.1	-80.7	10.30	-719.9	-63.9	8.15
6	-850.6	-790.2	-60.4	7.10	-794.5	-56.1	6.60	-796.8	-53.8	6.32
7	-927.6	-883.4	-44.2	4.76	-885.5	-42.1	4.54	-887.5	-40.1	4.32
8	-981.3	-969.6	-11.7	1.19	-961.6	-19.7	2.01	-958.3	-23.0	2.34
9	-1039.3	-1049.5	10.2	-0.98	-1044	4.7	-0.45	-1037.3	-2.0	0.19
10	-1112.4	-1123.4	11.0	-0.99	-1130.3	17.9	-1.61	-1135.9	23.5	-2.11
11	-1155.3	-1191.8	36.5	-3.16	-1199.5	44.2	-3.83	-1207.0	51.7	-4.48
12	-1166.9	-1255.1	88.2	-7.56	-1251.4	84.5	-7.24	-1248.7	81.8	-7.01

To further determine the advantages and disadvantages of the three prediction models, their accuracy was evaluated by comparing their MAPE, RMSE, SI and BIAS [35, 36] (Table 4). The smaller the four reference indicators, the smaller the actual predicted value error. The calculation formulas are as follows:

$$MAPE=\frac{1}{n}\sum_{t=1}^{n}\left|\frac{X_{t}-{\hat{X}}_{t}}{X_{t}}\right|$$ (16)

$$RMSE=\sqrt{\frac{1}{n}{\sum_{t=1}^{n}\left(X_{t}-{\hat{X}}_{t}\right)}^{2}}$$ (17)

$$SI=\frac{\sqrt{(1/n){\sum_{t=1}^{n}\left(\left(X_{t}-\bar{X_{t}})-({\hat{X}}_{t}-\bar{{\hat{X}}_{t}}\right)\right)}^{2}}}{\sqrt{(1/n)\sum_{t=1}^{n}{\hat{X}}_{t}}}$$ (18)

$$BIAS=\frac{\sum_{t=1}^{n}\left(X_{t}-{\hat{X}}_{t}\right)}{n}$$ (19)

where *X*_*t*_ is the measured value, ${\hat{X}}_{t}$ is the predicted value, $\bar{X_{t}}$ is the average of the measured value, $\bar{{\hat{X}}_{t}}$ is the average of the predicted value, and *n* is the number of sample data.

**Table 4:** Prediction accuracy test table.

Evaluation criterion of prediction performance	GM (1, 1)	FFBPNN	GM-FFBPNN
MAPE	9.09	7.61	7.39
RMSE	60.06	53.05	49.01
SI	1.07	0.42	0.06
BIAS	5.1	3.77	2.42

**Fig 11:**
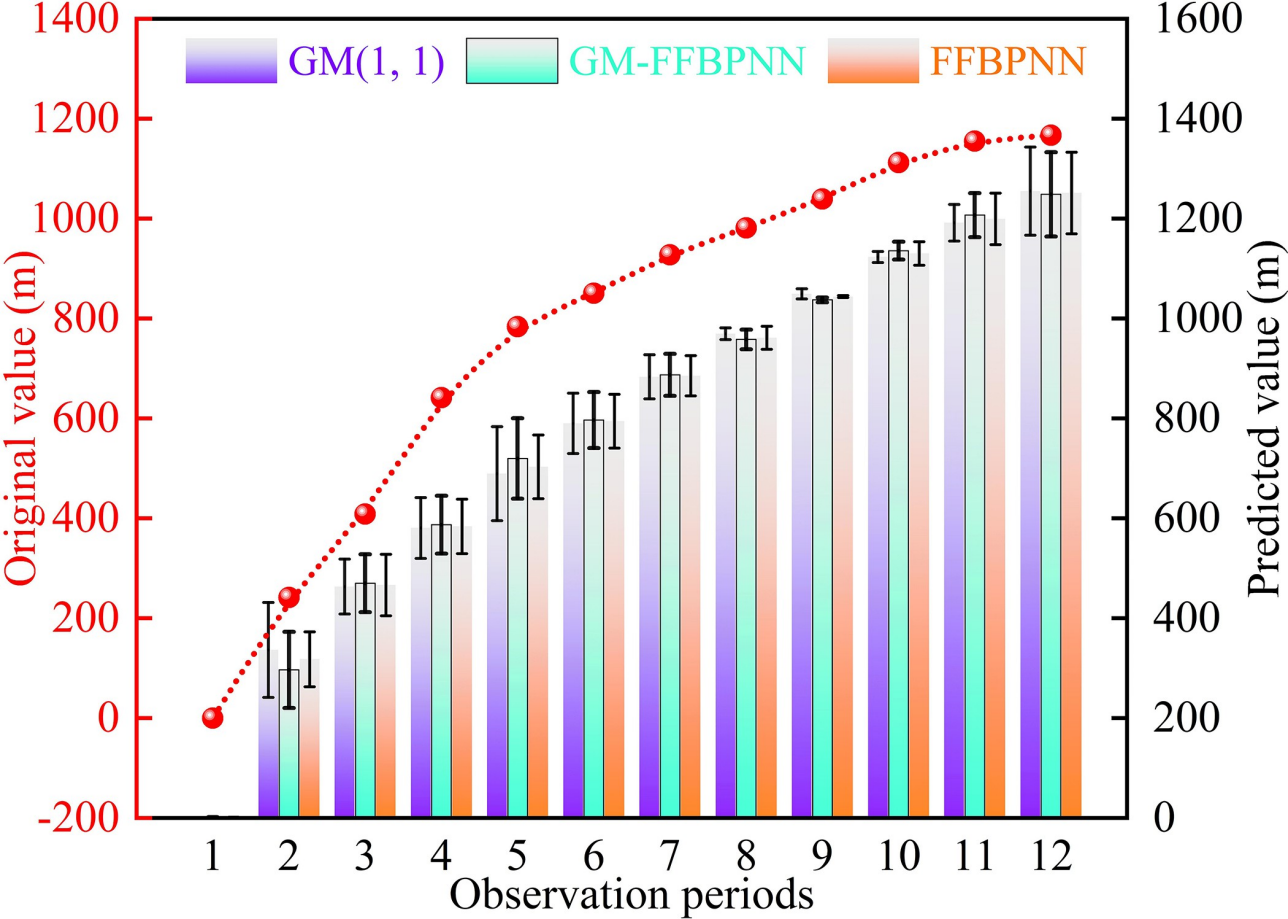
Comparison of predicted value and original value curve.

Table 4 shows that the mean absolute percentage and root mean square errors of the GM-FFBPNN were less than those of the GM (1,1), suggesting that the overall prediction effect of the GM-FFBPNN model was better than that of the GM (1,1). By comparing the prediction results of the two models (Table 3), we found that the predicted value of error compensation by the BP neural network was closer to the original value. Moreover, following preliminary training optimization, the residual error control was smaller, the maximum relative error decreased from −39.26% to −22.76%, and the non-uniformity of the GM (1,1) prediction error was reduced. To intuitively compare the development trend of the predicted values and original values of the three different models, change curves based on data in Table 4 were used (Fig 11).

Fig 11 shows that the development trend of the GM (1,1), FFBPNN, and GM-FFBPNN models were similar to that of the original value, although there was a small fluctuation that suggested that the three prediction models could better reflect the cumulative subsidence. However, from a macro perspective, the prediction curve of the GM-FFBPNN was closer to the original data curve, with a higher degree of fit, and the relatively moderate and smooth small dispersion degree was better than that of the GM (1,1) and FFBPNN. Thus, the proposed prediction model combined the advantages of the GM (1,1) and FFBPNN models. It not only effectively solved the problem of series with volatility and nonlinearity, but it also reduced the requirement for large sample datasets by the FFBPNN [37]. Hence, we demonstrated that the combined prediction model had more significant error optimization effects, a better stability performance, higher prediction accuracy, and more accurate and applicable data prediction abilities than the single prediction model.

### GM-FFBPNN model prediction after wavelet denoising

**Fig 12:**
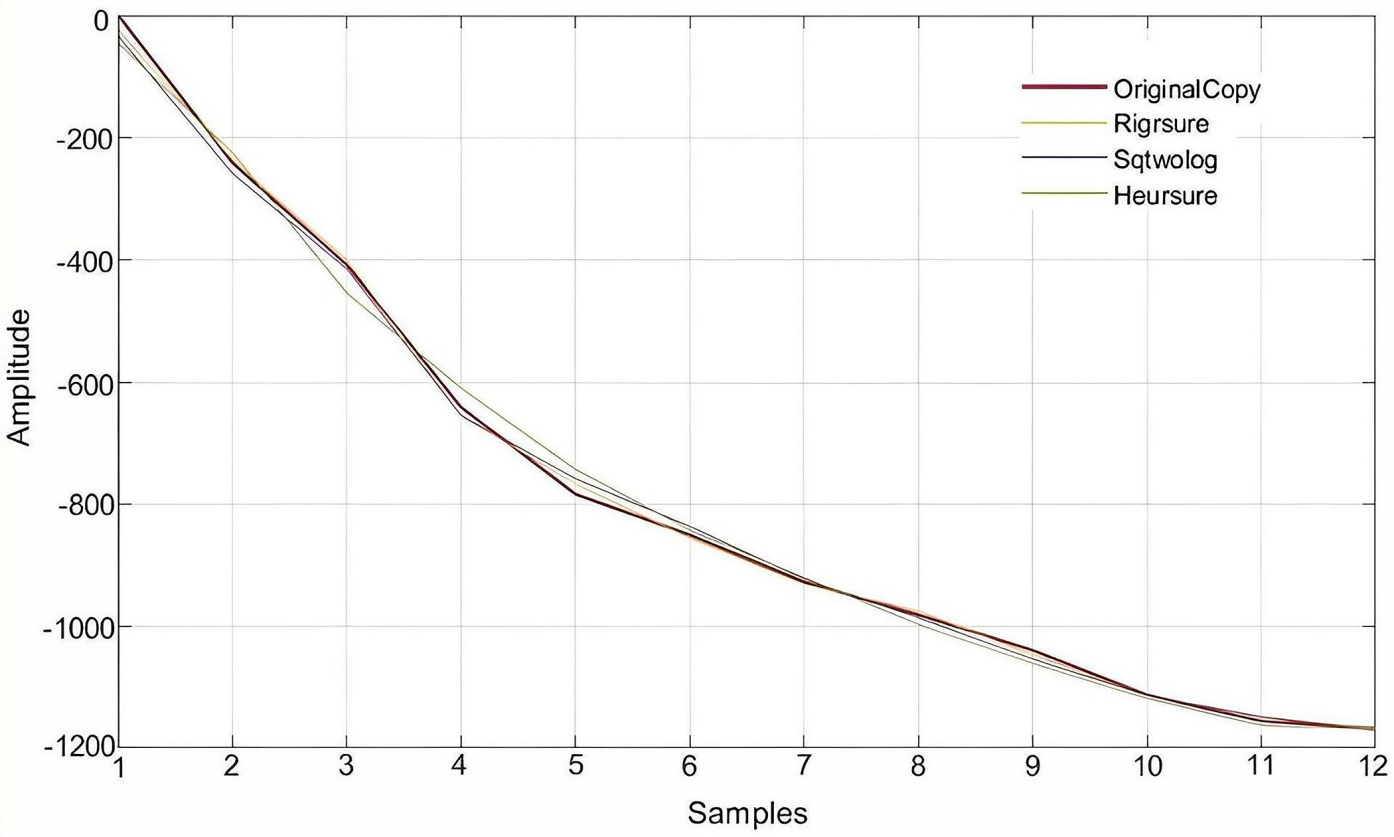
Comparison of four threshold denoising effect curves.

Using field application and monitoring point data analysis, the monitoring data of surface subsidence in the goaf is affected by several factors, resulting in forecasting errors when directly using raw data. To avoid this phenomenon and improve research efficiency, the wavelet function was introduced into the wavelet analysis toolbox of MATLAB software to pre-process the original data. In this study, the cumulative settlement of the N22 monitoring point was selected for wavelet threshold denoising analysis. To select the optimal threshold, a state in which all other factors remained constant was controlled by the control variable method, using the Rigrsure, Sqtwolog, Heursure, and Minimaxi methods to denoise the original data with unknown scale white noise, and different denoising effects were obtained [38]. The comparison curves of denoising effects using each of the four different threshold methods are shown in Fig 12.

The denoising effects differed in the chosen threshold. Subsequently, the reconstructed sinking curve was smoother and more stable, without oscillation and broken line phenomena. Wavelet threshold denoising improved and retained the original signal by removing the noise, thereby achieving the true function of N22 measuring point data denoising and providing a signal that was closer to real subsidence data. It was difficult to determine the effect of denoising only by curve comparison charts, therefore, the RMSE and signal-to-noise ratio (SNR) were used to further evaluate the wavelet denoising quality. Theoretically, the smaller the root mean square error, the greater the signal-to-noise ratio, the closer the denoising signal is to the original signal, and thus the better the denoising effect [39]. Because there were fewer than 32 observation periods at the monitoring point, the RMSE of the minimax threshold was zero and could not effectively denoise and the curve coincided with the original data curve. Therefore, only the denoising effects of the other three threshold methods must be compared, as shown in Table 5.

**Table 5:** Denoising quality evaluation table of different threshold methods.

Threshold mode	RMSE	SNR
Rigrsure	7.40	33.04
Sqtwolog	12.49	19.36
Heursure	21.93	10.17

**Fig 13:**
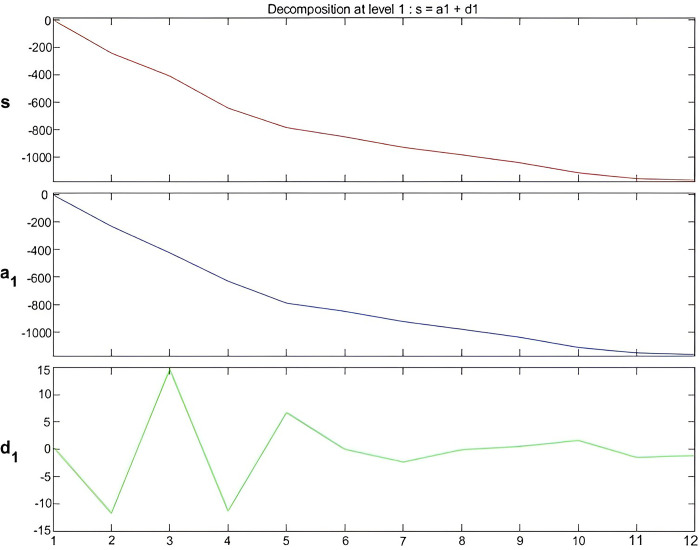
Approximate signal and detailed signal diagram after decomposition.

By comparing the evaluation index results of three different threshold methods, we concluded that the RMSE of the Rigrsure threshold function was 7.4 mm, which was lower than the RMSE of the other two threshold functions, and the SNR was 33.04, which was higher than the SNR of the two threshold functions. Therefore, the Rigrsure threshold function has a better denoising effect and more accurate prediction capability. Finally, Daubechies3 wavelet, Rigrsure threshold method and soft threshold principle are selected to denoise the cumulative settlement of monitoring points after one-layer decomposition. The sequence of each layer after denoising and decomposition of N22 point data is shown in Fig 13.

Regression analysis of the accumulated settlement denoised by wavelet analysis (Table 6) revealed that the denoising value was similar to the measured value, and the maximum relative error was 5.01% with no significant fluctuations. Additionally, the noise fluctuations in the last eight periods decreased gradually, the denoising values were more stable, and the relative errors were ± 1%, indicating a high degree of fitting. The real signal extracted by wavelet denoising was highly similar to the real settlement value, in line with the law of surface subsidence, further validating the reliability of the wavelet threshold denoising method. We found, through the prediction results of the GM-FFBPNN denoised by wavelet analyses, that the relative errors in the 12 periods were all controlled within ± 4%. The predicted value was roughly similar to the original value, and had a higher fitting degree, thus supporting the reliability and stability of this model.

**Table 6:** Cumulative settlement and predicted value of N22 monitoring points after denoising.

Number of monitoring periods	Original value (mm)	Regression analysis after denoising			GM-FFBPNN model after denoising		
		Denoising value (mm)	Residual error (mm)	Relative error (%)	Denoising value (mm)	Residual error (mm)	Relative error (%)
1	0.0	0.0	0.0	0.00	-3.2	3.2	**
2	-241.7	-229.6	-12.1	5.01	-232.2	-9.5	3.93
3	-408.5	-423.8	15.3	-3.75	-422.6	14.1	-3.45
4	-641.7	-628.9	-12.8	1.99	-619.3	-22.4	3.49
5	-783.8	-789.8	6.0	-0.77	-787.0	3.2	-0.41
6	-850.6	-850.6	0.0	0.00	-849.0	-1.6	0.19
7	-927.6	-925.1	-2.5	0.27	-922.0	-5.6	0.60
8	-981.3	-982.2	0.9	-0.09	-968.0	-13.3	1.36
9	-1039.3	-1040.4	1.1	-0.11	-1018.1	-21.2	2.04
10	-1112.4	-1115.1	2.7	-0.24	-1111.7	-0.7	0.06
11	-1155.3	-1153.2	-2.1	0.18	-1163.4	8.1	-0.70
12	-1166.9	-1165.5	-1.4	0.12	-1206.3	39.4	-3.38

**Fig 14:**
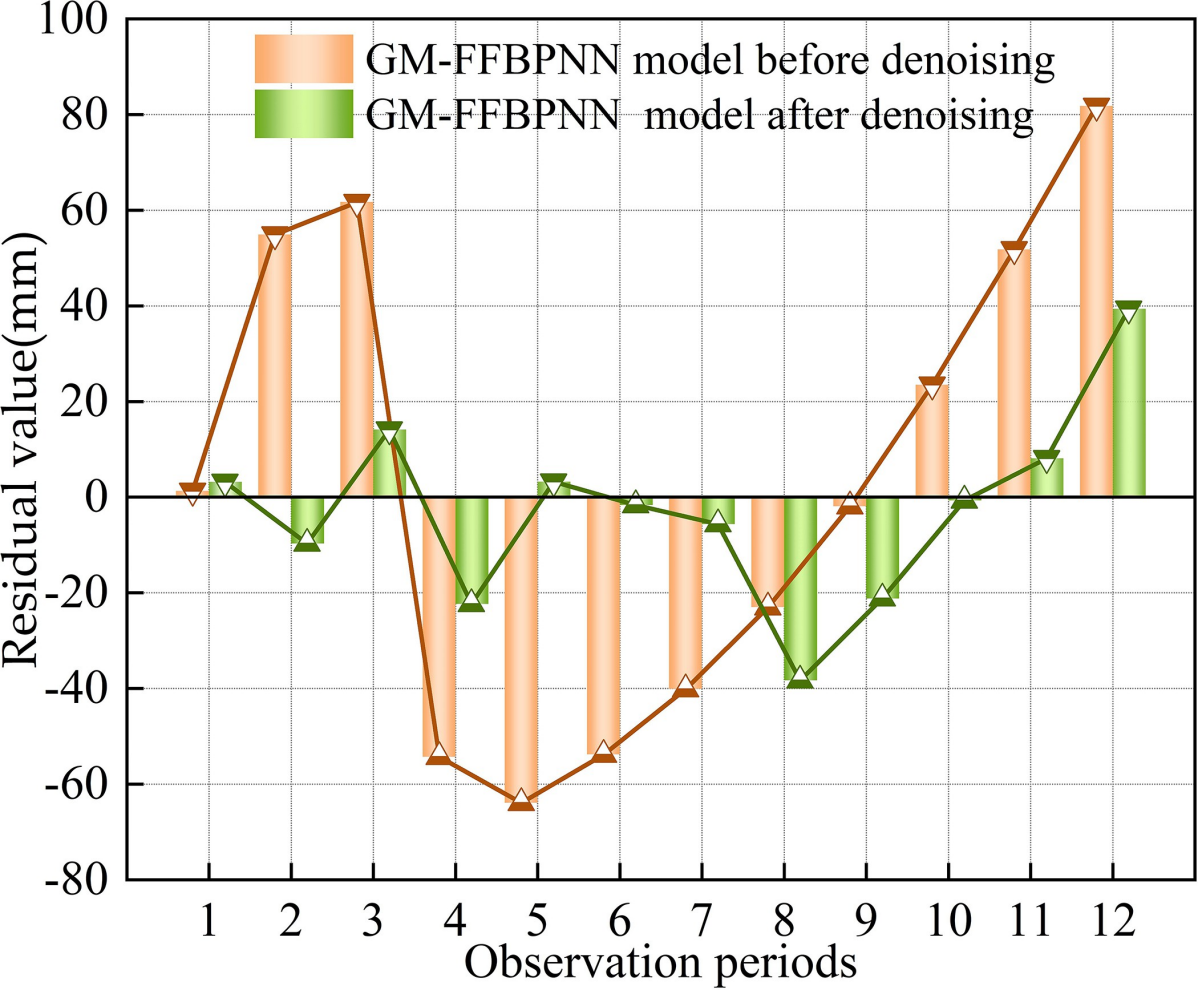
Comparison of residual value before and after denoising.

The data sequence was smooth after denoising by wavelet analyses, the errors of the learning sample of the GM-FFBPNN were adjusted, and the results were optimised, greatly improving the accuracy of the data and model [40]. The mean absolute percentage and RMSEs of the denoised GM-FFBPNN were 1.78% and 16.05 mm, respectively, which were significantly smaller than the error derived when using the original data for prediction. To verify the effects of wavelet denoising on the prediction accuracy of the GM-FFBPNN, the residual values of the prediction model before and after wavelet denoising were compared (Fig 14).

Though some residual values were larger after wavelet denoising, the residual values of most phase wavelets after denoising were less than those before denoising, suggesting that the noise signal in the measured data affected the prediction results of the combined model. Overall, the positive and negative trends of residual values were essentially identical before and after wavelet denoising; that is, the predicted values of the two combined models were both higher and lower than the measured values. Furthermore, denoising could only reduce the error of prediction but did not affect the overall prediction trend, suggesting that the error of the prediction results of the GM-FFBPNN based on the accumulated settlement after wavelet denoising was smaller and more stable than that when the original data was used to predict the settlement value directly, and provided results closer to the actual settlement value. There were also advantages over single models or other prediction methods [41–43].

The GM-FFBPNN model, which is based on wavelet denoising, combines the benefits of three prediction theories and achieves an organic combination of the advantages of various prediction methods. The accuracy and reliability of the modelling results were improved further through weight allocation, error correction, and structure optimization. The method has broad applicability in the analysis of changes characterized by volatility, randomness, and nonlinearity and it introduces a new method for predicting the settlement of small coal mine goafs or other similar projects. The theoretical basis can also be used to effectively monitor subsidence deformation in mining areas.

## Conclusion

This study explores practical engineering, combines the advantages of grey theory, neural network theory, and wavelet denoising theory, and develops a combined model in series to predict the surface residual settlement of goaf.

1. Using a mining area in Mentougou, Beijing as an engineering background, the general trend and settlement velocity of a surface residual settlement in a mined-out area of a small coal mine was analysed, in according with general subsidence law.
2. The GM (1,1) and FFBPNN model were established using MATLAB R2021a software to predict the surface residual settlement of goafs. The prediction accuracy of the two models was tested by evaluating their MAPE, RMSE, SI, and BIAS. The results show that the data fluctuation of a single model is large, and the error is non-uniform.

1. Combining the advantages of the GM (1,1) and FFBPNN, the GM-FFBPNN was developed in series for prediction. The results showed that the optimization error effect of the combined model was obvious, with MAPE, RMSE, SI, and BIAS decreasing to 7.39%, 49.01 mm, 0.06% and 2.42%, respectively. The combined GM-FFBPNN model has a higher prediction accuracy than a single model and was more closely related to the changing trend of the original data.
2. In order to further reduce error, the wavelet threshold denoising method was used to deal with noise in the original monitoring sequence. Thus, the denoising value obtained via reconstruction was applied to the GM-FFBPNN, greatly reducing the error value and improving the prediction accuracy. The prediction effect of the GM-FFBPNN model based on wavelet denoising meets engineering application needs, accurately reflects the goaf surface subsidence process, and has strong theoretical significance and application prospects.

## Supporting information

S1 File (ZIP)
